# An Integrated Approach to Defining Genetic and Environmental Determinants for Major Clinical Outcomes Involving Vitamin D

**DOI:** 10.1007/s40291-014-0087-2

**Published:** 2014-02-21

**Authors:** Antonio J. Berlanga-Taylor, Julian C. Knight

**Affiliations:** 1 CGAT, MRC Functional Genomics Unit, Department of Physiology, Anatomy and Genetics, University of Oxford, Oxford, OX1 3PT UK; 2 Wellcome Trust Centre for Human Genetics, Nuffield Department of Clinical Medicine, University of Oxford, Oxford, OX3 7BN UK

## Abstract

There is substantial genetic and epidemiological evidence implicating vitamin D in the pathogenesis of many common diseases. A number of studies have sought to define an association for disease with sequence variation in the *VDR* gene, encoding the ligand-activated nuclear hormone receptor for vitamin D. The results of such studies have been difficult to replicate and are likely to need to account for specific environmental exposures. Here, we review recent work that has begun to study the interactions between *VDR* gene polymorphisms, vitamin D blood levels, and complex disease susceptibility, notably in the context of major clinical outcomes. We highlight the challenges moving forward in this area and its importance for effective clinical translation of current research.

## Introduction

Vitamin D deficiency has been associated with a number of diseases but causality and molecular mechanisms remain poorly understood. Vitamin D is a secosteroid hormone classically known for its function in calcium metabolism and bone physiology with the first descriptions of rickets made in the 1600s by Whistler and Glisson [1]. The broader role of vitamin D in health and disease is highlighted by studies demonstrating its importance in inflammatory conditions and the adaptive and innate immune response. Epidemiological data, animal models, and genetic studies provide evidence of a role for vitamin D in diseases from cardiovascular disease (CVD) to type 1 diabetes mellitus and multiple sclerosis (MS) [2–5]. A recent study indicates that *VDR* polymorphisms may interact with circulating levels of vitamin D (25(OH)D) in determining major clinical outcomes, including a composite outcome of incident hip fracture, myocardial infarction (MI), cancer, and mortality [6]. Here, we focus on this recent evidence, review meta-analysis studies of *VDR* polymorphisms and disease, and highlight aspects relevant for future research.

### Sources and Metabolism of Vitamin D

Humans obtain vitamin D from skin exposure to ultraviolet B radiation (UVR) or through dietary intake. The metabolism is complex with many intermediate steps. UVR converts 7-dehydrocholesterol to pre-vitamin D_3_, which is immediately transformed through a heat-dependent process to vitamin D_3_ [7, 8]. Vitamin D_3_ (from UVR exposure) or D_2_ (dietary) is transported by chylomicrons and stored in adipocytes or transported to other tissues by the vitamin D-binding protein (*GC*) [8, 9]. Vitamin D-25-hydroxylase (*CYP2R1*) metabolizes vitamin D to 25-hydroxyvitamin D (25(OH)D), the form most commonly measured and used to determine vitamin D status in an individual [10, 11]. The active form of vitamin D, 1,25-dihydroxyvitamin D (1,25(OH)_2_D), is obtained by conversion of 25(OH)D by 25-hydroxyvitamin D-1α-hydroxylase (1-OHase; *CYP27B1*). *CYP2R1* and *CYP27B1* are expressed at high levels in the liver and kidney, respectively, although they are found in multiple tissues [10]. 1,25(OH)_2_D is catabolized by 1,25-dihydroxyvitamin D 24-hydroxylase (*CYP24A1*) to form calcitroic acid and is excreted in bile [10].

The actions of vitamin D are largely mediated through the vitamin D receptor (*VDR*). VDR is ligand activated by 1,25(OH)_2_D and as a heterodimer with the retinoid-X receptor (RXR) travels to the nucleus to bind vitamin D response elements. DNA-bound 1,25(OH)_2_D-VDR-RXR complexes regulate genome-wide transcriptional expression through incompletely understood mechanisms [12].

Although the most important source for vitamin D is exposure to sunlight, dietary supplementation is important for individuals at risk of deficiency. This is increasingly true for populations away from the equator, individuals with dark skin, those who use excessive sunscreen, and those who do not expose their skin to sunlight for religious or lifestyle reasons. As such, adequate guidelines for sun exposure and vitamin D supplementation remain necessary, particularly for at-risk groups.

### Genetic Determinants of Plasma Vitamin D Levels

The circulating form of vitamin D, 25(OH)D, is the most commonly measured and used form to describe an individual’s vitamin D status [9]. The definition of normal levels is contentious, particularly for non-skeletal functions. A recent report from the Institute of Medicine in the USA highlighted the need for rigorous evidence to be collected for non-classic effects of vitamin D and recommended plasma levels to be 50–150 nmol/L [13].

Recent studies have indicated that heritability of 25(OH)D in plasma can be high. It is estimated that ~25 % of inter-individual variability can be attributed to season of measurement, latitude, or intake [14]. A study in male monozygotic twins showed significant seasonal variation with a 15 nmol/L lower value during the winter than during the summer. It was estimated that 70 % of the variation during winter was explained by genetic factors, though in the summer, levels did not have a discernible heritable component [15]. Karohl and colleagues (2010) also confirmed a previous finding of significantly higher intra-class correlation between monozygotic twins compared with dizygotic twins [16].

Two recent genome-wide association studies (GWAS) found that common variants present in *CYP2R1, 7-DHCR, CYP24A1*, and in the vitamin D binding protein (*GC, DBP*), were associated with 25(OH)D blood levels [14]. These genetic markers and genotype combinations were associated with an increased risk of vitamin D insufficiency (<75 nmol/L), deficiency (<50 nmol/L), and severe deficiency (<20 nmol/L) [14, 17]. Interestingly, rs2282679 within the *GC* gene was also significantly associated with concentrations of GC protein with the minor allele associated with lower levels (Table 1) [14].

**Table 1:** Single nucleotide polymorphisms associated with vitamin D plasma levels by genome-wide association studies

References	Reported gene(s)	Strongest SNP	Risk allele frequency	*P* value	OR (*P* value)*
Ahn et al. [17]	*GC*	rs2282679	0.26	1.8E−49	1.83 (2.50E−8)
	*CYP2R1*	rs2060793	0.41	2.9E−17	0.89 (0.21)
	*NADSYN1, DHCR7*	rs3829251	0.19	3.4E−09	1.18 (0.11)
Wang et al. [14]	*GC*	rs2282679	0.29	1.9E−109	1.63 (3.5E−50)
	*CYP2R1*	rs10741657	0.40	3.3E−20	1.21 (4.1E−10)
	*NADSYN1, DHCR7*	rs12785878	0.23	2.1E−27	1.21 (9.4E−11)

*SNP* single nucleotide polymorphism, *OR* odds ratio

* Association of one risk allele with <25 nmol/L (Ahn et al.) and <75 nmol/L (Wang et al.)

### The Vitamin D Receptor

The discovery of VDR in the jawless primitive fish (lamprey) led to the conclusion that the vitamin D system originated before the development of calcified structures [18]. Ultraviolet irradiation in invertebrates, fungi, and plants generates vitamin D_2_ and thus vitamin D may have developed as a system to help prevent damage to nucleic acids and proteins [19]. VDR’s structural similarity to other nuclear receptors involved in bile acid and xenobiotic receptors raises the possibility that its initial importance related to detoxification and later evolved to calcium homeostasis and immunity [1]. The *VDR* gene is conserved across species including non-human primates, rodents, birds, and flies and it seems likely that it has been positively selected in humans [20]. It is thought that skin color evolved to address vitamin D synthesis [21]. Vitamin D deficiency leading to disorders such as rickets-induced pelvic fractures can have fatal consequences and may have driven selection to some extent [22].

**Fig. 1:**
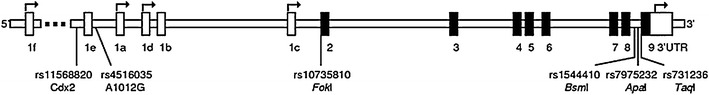
The human vitamin D receptor gene showing polymorphisms associated with disease. VDR spans 105 kb at chromosome 12q13.11. Studies independently investigating hip fracture, type 2 diabetes, rheumatoid arthritis (RA), MI, cancer, and other diseases such as MS, tuberculosis and type 1 diabetes have reported associations with *VDR* gene polymorphisms. dbSNP (rs) identifiers and nomenclature derived from restriction enzyme cleavage sites are shown in the context of the region chr12:46,524,238–46,525,237 on the negative strand (UCSC release hg18, March 2006). Exons marked *1f*–*1c* are largely non-coding and are indicated by open bars. Black bars indicate coding exons 2–9 with transcriptional start sites marked by arrows. Figure reproduced with permission from [26]

VDR is found in the nucleus as a homodimer in the absence of 1,25(OH)_2_D_3_. The VDR protein can bind to the three isoforms of RXR and acquires the active conformation when bound to 1,25(OH)_2_D_3_. More than 900 allelic variants have been described at the *VDR* locus (1000 Genomes Browser v. 3; *Homo sapiens*: GRCh37.p11, Chr 12 (NC_000012.11): 48.23–48.29 M). A small number of these have been studied with several disease associations reported. Importantly, loss-of-function mutations in the *VDR* gene cause hereditary vitamin D-resistant rickets type II [23, 24]. *ApaI* [rs7975232], *BsmI* [rs1544410], *FokI* [rs10735810], and *TaqI* [rs731236]) are single nucleotide polymorphisms (SNPs) in *VDR* that have been widely studied in relation to common disease, initially using restriction enzyme assays from which the nomenclature derives from (Fig. 1) [25–27]. Alleles identified by restriction sites are noted in upper (absence) and lower (presence) case respectively (e.g., ‘F’ or ‘f’ for FokI).

### Vitamin D Blood Levels, *VDR* Polymorphisms, and Major Clinical Outcomes

It is possible that genotypic effects are only evident under particular environmental contexts. A recent study showed that the combination of low circulating 25(OH)D levels and genotype increases disease risk for multiple outcomes [6]. Levin et al. [6] undertook a candidate gene approach to test whether common polymorphisms in vitamin D metabolism genes (*VDR*, *CYP27B1*, *CYP24A1*, *GC*, *LRP2*, and *CUBN*) interacted with circulating 25(OH)D and were associated with disease [6]. A composite outcome of incident hip fracture, MI, cancer, and mortality after a follow-up of 11 years in the discovery cohort was used. Independent replication was done using three additional cohorts from different populations with follow-ups of at least 7 years. The discovery cohort identified interactions between five SNPs and low circulating 25(OH)D levels. One SNP implicating *VDR* was replicated independently and low circulating 25(OH)D levels were associated with increased risk of the composite outcome with allelic dosage. Individuals with low circulating 25(OH)D levels and one minor allele at rs7968585 had a hazard ratio (HR) of 1.40 (95 % confidence interval [CI] 1.12–1.74) for the composite outcome while individuals with two alleles had a HR of 1.82 (95 % CI 1.31–2.54). Homozygous individuals for the major allele at rs7968585 showed no association.

This study is of special interest given the number of reported associations of vitamin D deficiency with disease. The magnitude and direction of the risks conferred by genetic variants were relatively consistent across populations and individual disease outcomes. However, heterogeneity was significant in the meta-analysis for two variants, and borderline non-significant for a third. Using a composite outcome may add statistical power but the biological reasoning is not immediately evident. Although vitamin D has many actions it would seem unlikely that it has a causal effect in multiple diseases with diverse pathophysiological mechanisms. The study by Levin et al. [6] is, to our knowledge, one of the first to use an analysis based on composite outcomes for *VDR* polymorphisms, disease association, and environmental interaction. Further studies assessing larger independent cohorts and interrogating homogenous outcomes will be required to confirm and refine these findings. Here, we set the results in a broader context by first highlighting recent systematic reviews and meta-analyses for each condition to contextualize these findings.

### Genetic Evidence of Association Involving the Vitamin D Receptor

#### Hip Fracture

Candidate gene studies testing the association of *VDR* polymorphisms and fracture have had mixed results. A meta-analysis suggested that there is a statistically significant association between *BsmI* and fracture risk [28]. By contrast *TaqI*, associated in the Levin et al. study, and other variants were not significant.

#### Type 2 Diabetes Mellitus

Studies showing an association between *VDR* polymorphisms and susceptibility to type 2 diabetes have been inconsistent. A recent meta-analysis to analyse the association of four well-studied *VDR* polymorphisms (*FokI*, *BsmI*, *ApaI*, and *TaqI*) with type 2 diabetes susceptibility found that the *FokI* polymorphism, but not *TaqI* or others, was significantly associated with an increased risk of type 2 diabetes in an Asian population [29]. None of the polymorphisms studied had significant associations in other populations.

A recent trial investigated the effect of *VDR FokI* polymorphism and vitamin D intake on glycemic status, lipid profiles, and inflammatory biomarkers in 140 diabetic subjects. It found that *FokI* (FF vs. ff) is associated with increased levels of plasma 25(OH)D and decreased levels of C-reactive protein (CRP) and interleukin (IL)-6 following 12 weeks of 1,000 IU per day [30]. The study concluded that individuals with the FF genotype, when compared with those with ff, had the largest increases in circulating 25(OH)D levels, as well as the largest decrements in CRP and IL-6. Findings from other studies, such as an inverse association between 1,25(OH)_2_D levels and chronic kidney disease in patients with type 2 diabetes that was significantly modified by *FokI* [31], support the notion that vitamin D and/or polymorphisms in the VDR may be important in diabetic physiopathology.

#### Rheumatoid Arthritis

A meta-analysis to determine the association between *VDR* polymorphisms (*BsmI*, *TaqI*, *FokI*, and *ApaI*) and RA susceptibility found that the *FokI* polymorphism was significantly associated in Europeans [32]. More recent studies have found several *VDR* polymorphisms associated with RA in different populations including South Asians and Caucasians [33], Tunisians [34], and Egyptians [35]. These studies have had small sample sizes with 200 or fewer cases each and replication is needed.

#### Myocardial Infarction

Few studies have investigated the role of *VDR* polymorphisms in MI susceptibility, although many observational studies have shown an association between low 25(OH)D levels and CVD. In patients aged <65 years, but not older individuals, there is evidence that *VDR* *BsmI* significantly associates with the presence of MI [36]. However, a new polymorphism in the binding site of the primers used for *BsmI* restriction polymorphism identification, causing failure of polymerase chain reaction amplification with a loss of the b allele in heterozygotes, may have confounded this and other studies [37].

#### Cancer

Multiple studies have associated *VDR* polymorphisms with cancer risk and progression, particularly for susceptibility to lung [38] and ovarian cancers [39]. Results have generally been mixed and meta-analyses have suggested positive results only for particular polymorphisms in certain populations. Significant associations have been reported in breast cancer (*FokI*, *BsmI*, *TaqI*, *ApaI*, *poly (A)*), prostate (*FokI*, *BsmI*, *TaqI*, *poly (A)*), skin (*FokI*, *BsmI*, *A-1210*), colorectal (*FokI, BsmI*), ovary (*FokI, ApaI*), and bladder (*FokI*) cancers, and in renal cell carcinoma (*TaqI*, *ApaI*) [40].

Associations were strongest for breast (*BsmI*, *FokI*) and prostate (*FokI*) cancers and malignant melanoma (*FokI*) on systematic review [40]. However, an updated meta-analysis for prostate cancer risk showed an association in Asian populations for *TaqI* [41], but *FokI* and *BsmI* in diverse populations showed no significant associations [42]. Lack of association has also been reported in colorectal cancer outcomes [43]. Meta-analysis of colorectal cancer risk studies showed increased risk associated with *BsmI* polymorphisms, but not *Cdx-2, FokI*, *ApaI*, or *TaqI*, in Caucasians [44]. Breast cancer association studies have also shown mixed results. A recent meta-analysis showed that *FokI* is associated with breast cancer risk in diverse populations while *ApaI* may be associated with Asian populations [45]. Other polymorphisms, such as *VDR poly(A)*, do not appear to be associated [46]. The VDR *Cdx2* polymorphism was found to be significantly associated with breast cancer susceptibility in Africans but not in Caucasians [47].

#### Genome-Wide Association Studies and Vitamin D

Several vitamin D metabolism genes have been implicated in GWAS of immunulogic, cancer, and inflammatory traits (Table 2). However, increased susceptibility of hip fracture, type 2 diabetes, RA, MI, and cancer other than lung has not been observed in association with SNPs in vitamin D metabolism genes. In particular, rs7968585, associated with these major clinical outcomes by Levin and colleagues [6], or variants in high linkage disequilibrium (rs10783215, rs7965281, rs4760733; *r* ^2^ > 0.9), have not been associated by GWAS. Differences in populations studied and criteria for phenotypic characterization among other issues may be responsible for this lack of replication. Large-scale studies in homogeneous populations with carefully selected endpoints are needed before definitive conclusions can be drawn.

**Table 2:** Genome-wide association studies implicating vitamin D metabolism genes

Implicated vitamin D gene	Name (function)	Associated trait	Strongest SNP-risk allele	*P* value	OR	95 % CI	References
*VDR*	Vitamin D receptor	Inflammatory bowel disease	rs11168249-C	8.0E−09	1.05	[1.024–1.084]	[48]
*CYP27B1*	25-hydroxyvitamin D_3_ 1-alpha-hydroxylase	Multiple sclerosis	rs703842-A	5.0E−11	1.23	NR	[49]
*CYP24A1*	1,25-dihydroxyvitamin D_3_ 24-hydroxylase, mitochondrial	Atopic dermatitis	rs16999165-T	2.0E−08	1.19	[1.12–1.26]	[50]
		Multiple sclerosis	rs2248359-G	3.0E−11	1.12	[1.1–1.13]	[51]
		Lung cancer	rs4809957	1.20E−08	1.13	[1.08–1.18]	[52]
*GC*	Vitamin D binding protein	Non-alcoholic fatty liver disease histology (other)	rs222054-C	1.0E−06	2.54	[0.17–4.91]	[53]
		Metabolite levels	rs1851024	1.0E−14	NR	NR	[54]
		Vitamin D levels	rs2282679	2.0E−14	NR	NR	[17]
		Vitamin D insufficiency	rs2282679	2.0E−109	NR	NR	[14]
		Vitamin D levels	rs2282679-C	2.0E−49	0.38	[0.32–0.44] unit decrease	[17]
*CUBN*	Cubilin*, renal and intestinal endocytic receptor involved in the absorption of various protein ligands including GC	Urinary albumin excretion	rs1801239-T	1.0E−11	0.08	NR	[55]
		MRI atrophy measures	rs6602175	3.0E−06	0.01	NR	[56]
		Folate pathway vitamin levels	rs1801222	3.0E−09	0.05	[0.030–0.070] unit decrease	[57]
		Folate pathway vitamin levels	rs11254363-A	1.0E−06	21.49	[7.71–35.27] pg/mL decrease	[58]
		Quantitative traits	rs10508517-A	6.0E−06	0.18	NR	[59]
*LRP2*	Megalin**, also an endocytic receptor, primarily found in kidney, which mediates reabsorption and metabolism of glomerular-filtered substances including uptake of GC	Anorexia nervosa	rs830998	9.0E−06	NR	NR	[14]
		Urate levels	rs2544390-C	4.0E−08	0.08	[0.053–0.111] unit decrease	[60]
*CYP2R1*	Vitamin D 25-hydroxylase	Vitamin D insufficiency	rs10741657	3.0E−20	NR	NR	[14]
		Vitamin D levels	rs2060793-A	3.0E−17	0.25	[0.15–0.35] unit increase	[17]
*DHCR7*	7-dehydrocholesterol (7DHC) reductase, catalyses conversion of 7DHC to cholesterol, thereby removing substrate availability for vitamin D synthesis	Vitamin D insufficiency	rs12785878	2.0E−27	NR	NR	[14]
		Vitamin D levels	rs3829251-A	3.0E−09	0.18	[0.12–0.24] unit decrease	[17]

*SNP* single nucleotide polymorphism, *OR* odds ratio, *CI* confidence interval, *MRI* magnetic resonance imaging, *NR* not reported

Data from http://www.genome.gov/gwastudies, accessed December, 27, 2013 except [52]

* Also known as intrinsic factor-cobalamin receptor

** Also known as low-density lipoprotein-related protein 2

### Epidemiological Evidence Involving Mortality, Vitamin D Levels, and Supplementation

A reverse J-shaped association between serum 25(OH)D levels and all-cause mortality was confirmed in a large, prospective, population-based study [61]. Adjusted relative risk (RR) with 95 % CI for all levels <60 nmol/L were significantly above 1 when compared with the reference group (75–99 nmol/L). Complementing this, a meta-analysis of vitamin D supplementation involving nearly 75,000 individuals from 32 trials concluded that vitamin D_3_ appears to decrease mortality from any cause (RR 0.94, 95 % CI 0.91–0.98), although mainly in elderly institutionalized women [62].

#### Autoimmune Diseases

Many studies have investigated the relationship between vitamin D levels, the risk of developing autoimmune diseases (AID), and if vitamin D supplements can modify disease course. A recent meta-analysis identified 219 articles with such outcomes and found that vitamin D deficiency (<75 nmol/L) is highly prevalent in both healthy and diseased individuals and that severe deficiency (<25 nmol/L) is associated with increased symptomatology. However, among interventional studies, it was found that only in type 1 diabetes does vitamin D supplementation reduce the risk of susceptibility with a dose-response effect [63].

#### Hip Fracture

Several trials have been carried out to assess the value of vitamin D supplementation in the prevention of hip fractures. A recent participant-level meta-analysis using 11 double-blind, randomized, controlled trials of oral vitamin D supplementation, with or without calcium, compared with placebo or calcium alone in those aged older than 65 years, concluded that supplementation with ≥800 IU daily appeared of benefit in the prevention of hip and non-vertebral fracture (HR, 0.70; 95 % CI 0.58–0.86, *P* value <0.001; actual intake analysis for hip fracture) [64].

#### Type 2 Diabetes Mellitus

Many observational studies and a number of clinical trials have been carried out to determine the relationship of vitamin D blood levels and supplementation with type 2 diabetes and results remain inconclusive. Khan and colleagues recently reported a systematic review and meta-analysis of prospective studies reporting association of circulating or dietary vitamin D levels with incident type 2 diabetes, metabolic syndrome, and insulin resistance. They found significant heterogeneity across studies and evidence of publication bias but concluded that vitamin D status at baseline in healthy adults is inversely associated with increased susceptibility to type 2 diabetes (RR 0.81, 95 % CI 0.71–0.92) and metabolic syndrome (RR 0.86, 95 % CI 0.80–0.92) [65].

A further systematic review and meta-analysis that included 15 trials assessed the effect of vitamin D supplementation on glycemic control and insulin resistance [66]. It found no significant improvement in fasting glucose, HbA(1c), or insulin resistance in those treated with vitamin D compared with placebo. Larger trials in homogeneous populations are needed to determine whether vitamin D supplementation is beneficial in glycemic control, insulin resistance, and type 2 diabetes prevention.

#### Rheumatoid Arthritis

Several studies have investigated the association between vitamin D intake and RA susceptibility as well as 25(OH)D levels and RA activity. A recent meta-analysis summarized these studies and found an association between total vitamin D intake and RA incidence without between-study heterogeneity. Individuals had a 24.2 % lower risk of developing RA when comparing highest vs. lowest vitamin D intake groups. Vitamin D levels were also inversely associated with RA activity [67].

Studies of vitamin D supplementation have not shown benefit so far in RA. Vitamin D supplementation of 800 IU per day appears ineffective in RA patients [68]. A large study to determine whether calcium plus vitamin D supplementation affects incidence of RA was carried out in the Women’s Health Initiative. More than 36,000 women were randomized to 1,000 mg calcium carbonate plus 400 IU of vitamin D_3_ daily or to placebo. In intention-to-treat analyses, no differences were observed in RA incidence between treatment groups. Trials assessing higher doses are required before conclusions can be reached [69].

#### Myocardial Infarction

Multiple studies have shown a high prevalence of vitamin D deficiency in patients with CVD and an association of vitamin D deficiency with higher mortality [70, 71]. Vitamin D deficiency is also associated with an increased incidence of cardiovascular risk factors including hypertension, hyperlipidemia, MI, stroke, chronic kidney disease, and diabetes (reviewed in [72]). A recent prospective evaluation in multi-ethnic populations free of known CVD at baseline showed that white or Chinese, but not black or Hispanic, individuals with low circulating 25(OH)D levels were at significantly increased risk of MI, angina, cardiac arrest, or death from CVD [73]. Severe vitamin D deficiency in patients with acute coronary syndromes appears to be significantly and independently associated with in-hospital death [74].

Initial clinical trials to test whether supplementation with vitamin D is beneficial in patients with CVD have shown benefit [70]. Both low circulating 25(OH)D levels and high plasma renin activity are associated independently with poor prognosis in patients with chronic heart failure [75, 76]. A recent open-label, blinded, endpoint, phase II trial in cardiac heart failure patients showed that vitamin D_3_ supplementation lowers plasma renin activity using 2,000 IU daily [77]. A large randomized trial in healthy postmenopausal women from the Women’s Health Initiative showed that calcium (500 mg) plus vitamin D (400 IU daily) supplementation neither increased nor decreased coronary risk [78].

Strong and independent associations of circulating 25(OH)D levels with cardiovascular mortality have been shown but causality and benefit from supplementation remain unresolved.

#### Cancer

In vitro and animal models show strong evidence of anti-proliferative and pro-apoptotic effects in cancer cells following calcitriol stimulation [79]. Many observational studies have associated low circulating levels of 25(OH)D with cancer susceptibility, particularly for colorectal cancer [80]. A recent systematic review and meta-analysis of longitudinal studies showed an inverse association of total cancer incidence with circulating 25(OH)D levels [81]. Total cancer mortality was also assessed but studies showed significant heterogeneity; however, other studies have not shown an association. The Cohort Consortium Vitamin D Pooling Project of Rarer Cancers did not show a link between higher levels of vitamin D and reduced risk of cancer [82]. Clinical trials have shown mixed results and further studies are needed [83, 84].

### Low Levels of 25-Hydroxyvitamin D and Genotype in Major Clinical Disease Risk

The study by Levin et al. [6] showed an interaction between rs7968585 (implicating *VDR*) and low 25-hydroxyvitamin D concentration with increasing risk of the clinical composite outcome for individuals heterozygous and homozygous for the risk allele. It would seem reasonable to hypothesize that SNPs associated by GWAS with circulating 25(OH)D levels could serve as proxies, interact with *VDR* SNPs to determine disease risk, or be independently associated by GWAS with disease. GWAS have been carried out for these major disease outcomes and have not shown an association for the most highly significant SNPs associated with vitamin D levels (Table 2). Epistasis between SNPs found in or near vitamin D metabolism genes has been shown [14]. Wang et al. [14] combined the three confirmed variants and found that individuals with a genotype score in the highest quartile were at increased risk of having lower 25(OH)D circulating levels (<75 nmol/L and <50 nmol/L) compared with the lowest quartile. Integrating this information with disease phenotypes, as suggested by the approach used by Levin et al. [6], may reveal important insights and could help identify individuals at greater risk of disease susceptibility.

The Tromsø Study, a prospective assessment of more than 9,000 subjects in Norway, could not however support or exclude a causal relationship between SNPs associated with low 25(OH)D levels by GWAS and disease [85]. The study followed more than 9,000 individuals for 13 years with the endpoints MI, type 2 diabetes, cancer, death, or a randomly selected control group with genotyping for 17 SNPs related to serum 25(OH)D levels (those initially identified in [14, 17]). *VDR* variants were not included as these had not been consistently shown to affect circulating 25(OH)D levels. Few other studies have assessed interactions of this nature and more research is needed.

### Future Studies Assessing Vitamin D–Gene Interactions in Disease

#### Study Design and Analytical Considerations

Advances in the determination of genetic risk are greatly improving our capacity to detect gene–environment interactions (GEI). Although a detailed discussion of the study of GEI is beyond the scope of this review, we will mention several important aspects. Achieving sufficient power and appropriate study design are particularly challenging. Four designs have traditionally been used: family based, case-control, cohort, and more recently, case-only [86, 87]. Obtaining sufficiently large samples sizes remains the biggest obstacle in all. Susceptibility to population stratification bias, recall bias, survivor bias, prospectively collected samples for biomarker measurement, and sample size are particularly relevant factors [88]. Analysis and interpretation, especially for genome-wide designs, are rapidly becoming bottlenecks as sample sizes and data sets increase.

Case-only designs, where differences in the prevalence of the exposure between genotype-positive vs. genotype-negative cases would indicate an interaction, have recently gained popularity as they require smaller sample sizes and bias does not appear to be common in practice [86]. Case-control studies in clinical settings for gene–drug interactions have also been performed recently. The first large randomized trials of the clinical utility of genotype-guided dosing for vitamin K antagonists disappointingly showed marginal or non-usefulness for initiation of anticoagulant therapy [89–91]. Although results were not encouraging, studies of this nature will likely be more common in the future as we gain a better understanding of GEI and their clinical relevance.

Mendelian randomization has served as a useful method to interrogate GEI such as in hypertension risk and alcohol intake [92] and incidence of coronary events and low-density lipoprotein levels [93]. More recently, large studies such as the National Cancer Institute Breast and Prostate Cancer Cohort Consortium have provided examples of candidate gene and genome-wide GEI analysis in prostate cancer and steroid hormones including vitamin D [94, 95]. Contrary to experimental evidence, the study by Mondul et al. [94] found that variants related to lower levels of circulating 25(OH)D may actually be associated with a decreased risk of aggressive prostate cancer.

The study by Levin et al. [6] has several strengths and provides a useful starting point for discussion of study design in genetic association and 25(OH)D analysis. Replication of findings in different and independently recruited and ascertained populations greatly increases confidence by reducing the likelihood of false-positive results. Equally, independent analysis followed by meta-analysis provides more robust methodology. Stringent multiple testing correction is necessary. The use of a composite outcome may increase statistical power, although heterogeneity in phenotypic characterization across cohorts and likely underlying biologic differences in patho-physiology may counter this. Lifestyle factors, comorbidity, time from 25(OH)D status measurement, season of measurement, and body mass index need to be considered when analyzing disease associations involving circulating 25(OH)D levels.

Study outcomes and power must be clearly determined a priori to further avoid the likelihood of false-positive and false-negative results. Previous studies can aid in estimating effect size and sample sizes required. Initial recruitment of a homogeneous population, particularly in terms of genetic structure, is essential for successful genetic studies. Bias in reporting, level of diagnostic ascertainment, and known comorbidity are important factors that require careful consideration and attention when characterizing phenotypes for GWAS. The choice of platform to determine genotypes may need expert advice. For gene- or pathway-focused studies, analysis of published data, haplotype structure of the population of interest, and available functional evidence in a disease-relevant context would facilitate design and interpretation of results.

Finally, standardized methods of measurement of circulating 25(OH)D levels across individuals and cohorts can reduce artificial variation. Although 25(OH)D status is stable across extended periods of time, repeated measurements may be necessary. Despite intense debate, the report by the Institute of Medicine (USA) in 2011 [13] established thresholds for vitamin D levels that may aid comparisons across cohorts and diseases.

#### Future Clinical Applications: SNP Profiling for Identification and Treatment of At-Risk Individuals?

The number of studies associating low levels of circulating 25(OH)D with disease risk have raised calls for public health intervention. Should all individuals at particular latitudes be supplemented with vitamin D through diet? This question is far from trivial. Although proponents may eventually be proved right, conclusive evidence for the causal involvement and benefit from supplementation of vitamin D in non-classical vitamin D-associated diseases is still needed.

Interventions for individuals at greater risk could come sooner and may be more appropriate given the current evidence. Modification of lifestyle factors, while considering changes in risk for other diseases (i.e., increased sun exposure in fair-skinned individuals), and seasonal and dietary supplementation could be considered. Testing before treating, particularly in light of increased mortality at increased levels of circulating 25(OH)D [61], may be suitable in certain patient groups.

Identification of individuals at increased disease risk by genotype is a promising area. Evidence is currently insufficient to recommend this approach in vitamin D-related diseases but the study by Levin et al. [6] further raises this issue. Interestingly, Martineau et al. [96] showed in a randomized, controlled clinical trial in patients being treated for tuberculosis that vitamin D supplementation significantly decreased time to mycobacterium sputum clearance in individuals carrying rs731236 (*TaqI* [tt], recently merged with rs7968585, the variant identified by Levin et al. [6]).

## Conclusions and Future Perspectives

Observational data clearly associate vitamin D with many diseases and initial molecular evidence of the involvement of vitamin D through the action of VDR exists for some of these [97]. A causal role for vitamin D, other than in bone disease, appears to be more likely in MS and type 1 diabetes. Strong evidence for other diseases is not yet available.

Candidate gene association studies have many advantages but have largely suffered from inconsistency and lack replication. GWAS is a powerful approach to identify disease-associated genetic variants and hundreds of phenotypes have now been catalogued. There has been a lack of association of *VDR* polymorphisms in GWAS although other genes important in vitamin D metabolism, such as *CYP24A1* and *CYP27B1*, have been strongly implicated.

Benefit from vitamin D supplementation is yet to be systematically tested and future studies should address this through careful study design with a particular focus on at-risk individuals. Recent clinical trials of vitamin D supplementation have mostly used small numbers of individuals and have been underpowered showing inconsistent results.

Vitamin D–gene interactions are likely to be important in disease and require further study. *VDR* polymorphisms may play an important role in determining disease risk and further studies may need to take these variants, and those shown by GWAS to be important in determining circulating 25(OH)D levels, into careful consideration. Epistasis between variants that determine vitamin D blood levels and *VDR* SNPs may also be relevant and require investigation. Studies testing the interaction between variants associated with vitamin D blood levels, *VDR* polymorphisms, circulating 25(OH)D levels, and disease susceptibility may shed further insight into patho-physiological mechanisms and could serve to identify biomarkers. Genetic variant analysis as a means to identify patients at risk is a promising approach. However, it is premature to recommend genotyping *VDR* in a clinical setting and many more studies are needed before conclusive evidence can be reached. It seems likely that both common and rare variants in vitamin D metabolism genes play a role in disease. Further research addressing this question could yield additional insight that is clinically translatable.
